# Acid Sphingomyelinase, a Lysosomal and Secretory Phospholipase C, Is Key for Cellular Phospholipid Catabolism

**DOI:** 10.3390/ijms22169001

**Published:** 2021-08-20

**Authors:** Bernadette Breiden, Konrad Sandhoff

**Affiliations:** 1 Independent Researcher, 50181 Bedburg, Germany; bbreiden@gmx.de; 2 Membrane Biology and Lipid Biochemistry Unit, LIMES Institute, University of Bonn, 53121 Bonn, Germany

**Keywords:** acid sphingomyelinase deficiency (ASMD), Niemann-Pick disease, lysosomal phospholipase C, sphingomyelin, membrane lipids, ASM inhibitors, regulation, lipid accumulation, topology, depression

## Abstract

Here, we present the main features of human acid sphingomyelinase (ASM), its biosynthesis, processing and intracellular trafficking, its structure, its broad substrate specificity, and the proposed mode of action at the surface of the phospholipid substrate carrying intraendolysosomal luminal vesicles. In addition, we discuss the complex regulation of its phospholipid cleaving activity by membrane lipids and lipid-binding proteins. The majority of the literature implies that ASM hydrolyses solely sphingomyelin to generate ceramide and ignores its ability to degrade further substrates. Indeed, more than twenty different phospholipids are cleaved by ASM in vitro, including some minor but functionally important phospholipids such as the growth factor ceramide-1-phosphate and the unique lysosomal lysolipid bis(monoacylglycero)phosphate. The inherited ASM deficiency, Niemann-Pick disease type A and B, impairs mainly, but not only, cellular sphingomyelin catabolism, causing a progressive sphingomyelin accumulation, which furthermore triggers a secondary accumulation of lipids (cholesterol, glucosylceramide, GM2) by inhibiting their turnover in late endosomes and lysosomes. However, ASM appears to be involved in a variety of major cellular functions with a regulatory significance for an increasing number of metabolic disorders. The biochemical characteristics of ASM, their potential effect on cellular lipid turnover, as well as a potential impact on physiological processes will be discussed.

## 1. Introduction

Amphiphilic phospholipids (PLs) and proteins are the main and crucial building blocks of eukaryotic cellular membranes [1,2]. Besides the dominating glycero-PLs, mammalian cell membranes also contain a varying share of sphingolipids, cell type specific glycosphingolipids, and the sphingo-PL sphingomyelin (SM). Their major catabolic pathways occur within the lysosomal system, mostly catalyzed by rather unspecific hydrolases, lipases and glycosidases, sometimes supported by cofactors such as lipid binding proteins (saposins (Saps) and GM2 activator protein (GM2AP)). In the acidic environment of endosomes and lysosomes, complex lipids and other amphiphilic macromolecules are degraded on intralysosomal luminal vesicles (ILVs), and their components are released into the cytosol of the cell as fuel for energy metabolism and substrates for biosynthetic pathways. Whereas different glycero-PLs can be catabolized by rather substrate-unspecific lipases in alternative pathways, sphingolipids of mammalian cells are degraded in a stepwise manner without an effective bypass in human lysosomal catabolism. Therefore, an inherited defect of any catabolic step causes a progressive accumulation of the undegradable sphingolipid substrates, triggering a sphingolipid storage disease. Long before the molecular and metabolic basis of the inherited storage diseases had been elucidated, their clinical picture and fatal pathology was described. Few genotype–phenotype correlations have been observed recently in patients with Niemann-Pick disease types A and B [3]. In 1914, pediatrician Albert Niemann in Berlin published a report on a female infant who suffered from a progressive and severe hepatosplenomegaly, passing away at 18 months of age. Her visceral organs were full of foam cells like those in Gaucher disease [4]. Ludwig Pick identified further cases of similar etiology, clearly differentiating them from Gaucher disease [5]. Later, the disease became known as Niemann-Pick disease, a typical lysosomal lipidosis.

By a chemical analysis of postmortem brain tissue, Ernst Klenk identified the amphiphilic lipid SM as the main and primary storage compound in Niemann-Pick disease [6]. It is a major component of the outer leaflet of cellular plasma membranes, especially of neurons. It was first isolated and characterized in 1884 by Johann L. W. Thudichum—who was trained in chemistry by Justus Liebig in Giessen, Germany [7]. A SM-cleaving hydrolase, the SM phosphodiesterase also called acid sphingomyelinase (ASM, E.C. 3.1.4.12) was discovered by Shimon Gatt in 1966 [8] and its deficiency identified by R. O. Brady as the cause of the progressive SM accumulation in Niemann-Pick disease [9]. Autosomal recessive Niemann-Pick disease caused by ASM deficiency is a rare lysosomal storage disorder with a broad disease spectrum that includes chronic visceral (type B) and neurovisceral forms (intermediate type A/B) as well as the infantile, rapidly progressive fatal neurovisceral disease (type A) with type B being the most prevalent [10]

More recently, Niemann-Pick disease type A and B was renamed as ASM deficiency or ASMD to clearly differentiate the underlying pathology from Niemann-Pick disease type C which is caused by an intracellular cholesterol transport defect leading to the accumulation of cholesterol and SM but not by ASM deficiency [11].

The purpose of this comprehensive review is to summarize the main features of human ASM, its biosynthesis, processing and intracellular trafficking, its structure and proposed mode of action at the surface of PL-substrate carrying intraendolysosomal vesicles. Its PL-cleaving activity regulation by membrane lipids and sphingolipid activator proteins, lipid-binding proteins with a broad substrate specificity, will be highlighted. The majority of ASM literature implies that ASM specifically hydrolyses SM as a substrate to generate ceramide and phosphocholine and ignores its ability to degrade further substrates. In vitro experiments with radiolabeled PLs show that more than twenty different PLs are cleaved by ASM including some minor but functionally important PLs such as the unique lysosomal lysolipid bis(monoacylglycero)phosphate (BMP) and the cellular growth factor ceramide-1-phosphate (Cer1P).

The recessively inherited ASMD mainly leads to impaired cellular SM catabolism causing progressive SM accumulation in the cellular membranes. Furthermore, ASM appears to be involved in a variety of major cellular, endolysosomal regulatory functions for an increasing number of metabolic disorders. The biochemical characteristics of ASM, their potential effect on cellular lipid turnover, as well as their potential impact on physiological processes will be discussed.

## 2. Biosynthesis, Cellular Processing, Trafficking and Structure of Human ASM

**Figure 1:**
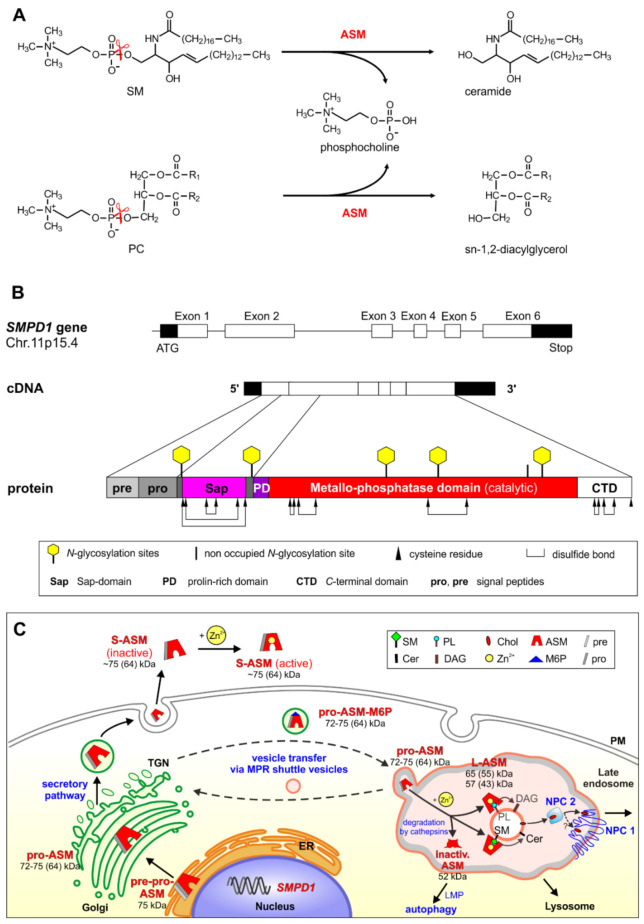
(**A**) ASM acts as an endolysosomal phospholipase C that cleaves off the phosphocholine residue from sphingomyelin and phosphatidylcholine. (**B**) Schematic illustration of the gene structure (exons represented in boxes), cDNA and processing structure of human ASM. The gene *SMPD1* of the ASM has been mapped to the chromosomal region 11p15.4. Mature human ASM contains several domains: a signal peptide (amino acids 1–46), a Sap-domain (amino acids 89–165), a proline-rich domain (amino acids 166–198), the catalytic metallo-phosphatase domain (amino acids 199–461), and the *C*-terminal domain (amino acids 462–629). *N*-glycosylation sites and disulfide bridges are shown. (**C**) Topology of ASM processing. Pro-ASM is generated from pre-pro-ASM within the Golgi and is transported after mannose-6-phosphorylation by mannose-6-phosphate receptor mediated vesicle transfer to the endolysosomal compartments (L-ASM) or exported by the secretory pathway (S-ASM). In the endolysosomal compartment, the pro-ASM matures to the active forms. If L-ASM cannot interact with the intraendolysosomal luminal vesicles, the enzyme is degraded by cathepsins (inactivated ASM). This process leads to higher levels of sphingomyelin, which may disturb membrane homeostasis and cause lysosome membrane permeabilization (LMP), which triggers cell-death by apoptosis and apoptosis-like pathways. The molecular mass given in brakes is the deglycosylated form. ASM: acid sphingomyelinase, Cer: ceramide, Chol: cholesterol, CTD: *C*-terminal domain, DAG: diacylglycerol, ER: endoplasmic reticulum, L-ASM: lysosomal ASM, LMP: lysosome membrane permeabilization, M6P: mannose-6-phosphate, MPR: mannose-6-phosphate receptor, NPC 2: Niemann-Pick disease protein C type 2, PC: phosphatidylcholine, PD: proline-rich domain, PL: phospholipid, PM: plasma membrane, pre-pro-ASM: pre-pro form of ASM, pro-ASM: pro form of ASM, Sap: Sap-domain, S-ASM: secretory ASM, SM: sphingomyelin, TGN: trans Golgi network.

ASM is a major cellular phosphodiesterase also known as phospholipase C (PLC), cleaving the phosphodiester bond of its PL substrates as shown in Figure 1A for the sphingo-PL SM and the glycero-PL phosphatidylcholine (PC) at low pH values.

After several unsuccessful attempts in various laboratories, the secretory form of ASM was eventually purified to homogeneity by factor 25.000 from human urine, collected from patients suffering from a cancer breakthrough or heavy traumatic organ damage [12,13]. Its sequencing allowed the decoding of the cDNA-encoding ASM [14,15], which was needed to analyze the molecular basis of ASMD also called Niemann-Pick disease, type A and B [16,17], and to identify the structural organization of the complete gene that encodes ASM (Figure 1B) [18]. Human ASM is encoded by the *SMPD1* gene located in the chromosomal region 11p15.4. The full-length cDNA of ASM contains an open reading frame of 1890 bp encoding 629 amino acids. The mature enzyme was shown to be a monomeric glycoprotein containing a protein core of 64 kDa. It has eight disulfide bridges [19] and five of the six *N*-glycosylation sites are occupied [20] (Figure 1B).

The availability of ASM-specific polyclonal antibodies allowed the elucidation of the intracellular processing of human ASM by multiple proteolytic steps (Figure 1C) [21,22]. ASM is first synthesized in the endoplasmic reticulum as a 75 kDa pre-pro-enzyme, which is rapidly processed after signal peptide cleavage to the 72 kDa pro-ASM in the endoplasmic reticulum-Golgi complex. Thereafter, the pro-ASM is transported to endolysosomal compartments or is exocytosed to the extracellular space by the secretory pathway (Figure 1C). The intracellular transfer of ASM and many other lysosomal hydrolases from the trans-Golgi network (TGN) to late endosomes and lysosomes depends on the mannose-6-phosphate receptor (M6PR) (Figure 1C), whereas sortilin provides an alternative route for ASM and the lipid binding proteins prosaposin and GM2AP to the phagosome [23,24,25,26]. The mature lysosomal ASM is generated by *C*-terminal proteolytic processing [27]. After cell treatment with cationic amphiphilic drugs (CADs), ASM cannot any longer attach properly to the intraendolysosomal luminal membranes and is rapidly degraded to an inactive form (52 kDa) [28] by cathepsins [29,30,31]. A second pathway leads to the secretion of ASM into the extracellular space. The major active form secreted has a size of ~75 kDa, a minor one of 57 kDa [22]. Both forms of ASM require zinc ions for activity [32].

Mature human ASM contains several domains: a signal peptide (amino acids 1–46), a Sap-domain (amino acids 89–165), a proline-rich linker domain (amino acids 166–198), the catalytic metallo-phosphtase domain (amino acids 199–461), and the *C*-terminal domain (amino acids 462–629) [33]. Whereas most sphingolipid hydrolases need the help of a lipid binding protein to reach a physiological turnover of (glyco)sphingolipids in the lysosomal compartment (GM2AP or one of the Saps A, B, C or D), the polypeptide of the ASM contains an *N*-terminal Sap-domain, which maintains basic SM-cleaving activity even in the absence of external sphingolipid activator proteins [34]. Therefore, SM hydrolysis is not significantly affected by the absence of Saps in prosaposin deficient patients and mice [35]. The structure of the Sap-domain of ASM is similar to that of Saps A–D [33]. These proteins can bind to membranes and undergo a conformational change [36,37] from a closed conformation to an open form that allows interaction with anionic charged membranes [38,39].

The catalytic metallo-phosphatase domain contains a binuclear zinc-center at its active site that binds SM with its ceramide-phosphate moiety and triggers a nucleophilic attack of an activated water molecule to split the phosphodiester bond. The catalytic domain contains a binuclear zinc-center at its active site that binds SM with its ceramide-phosphate moiety and triggers a nucleophilic attack of an activated water molecule to split the phosphodiester bond. Its cooperation with the flexible Sap-domain is essential for activity [33,38,39] (Figure 1B). This notion is supported by a complete loss of ASM activity in an infantile patient having a point mutation in its Sap-domain. The patient developed a severe form of Niemann-Pick disease type A with hepatosplenomegaly, developmental delay, and rapid neurological deterioration [40]. His fatal lipidosis emphasizes the essential function of the SAP domain for cellular SM turnover. Apparently, the Sap-domain of ASM allows a basal SM turnover in the lysosome even in the absence of any external Saps [41,42]. A basal lipid turnover is also achieved by other hydrolases acting on small membrane-bound sphingolipids in the absence of a sphingolipid activator protein in vitro, like glucosylceramide hydrolysis by β-glucocerebrosidase encoded by *GBA 1* [43] and ceramide degradation by acid ceramidase [44]. However, the turnover achieved in vivo in the absence of Saps is too low to prevent lipid storage [34]. In contrast, the lysosomal breakdown of the complex membrane-bound gangliosides GM1 and GM2 depends heavily on their lipid binding cofactors, GM2AP and Sap B [45,46,47].

## 3. ASM, an Endolysosomal and Secretory Phospholipase C, Cleaving Membrane Lipids

Most lysosomal hydrolases are unspecific (promiscuous) enzymes and degrade in vivo and in vitro soluble substrates such as oligosaccharides, and insoluble molecules such as membrane-bound lipids and sphingolipids as well as synthetic and fluorescence generating soluble substrates in vitro, often used to assay the overall activity of a hydrolase in biological samples. This overall activity, however, is not identical with the lipid cleaving activity of the hydrolase. Its lipid splitting activity is often heavily regulated in a complex manner by features of the lipid-substrate carrying vesicle membrane, by:

(a) the surface potential (measured by zeta potential) of the liposomal vesicles [48], modelling the ILVs of the lysosomal compartment,
(b) the curvature of the membrane vesicles (e.g., the ILVs) [44],
(c) the hydrolysis stimulating lipid binding proteins (the sphingolipid activator proteins) [49],
(d) stimulating lipids, such as BMP [43,48,50,51], and
(e) inhibiting lipids such as SM [43,46,50].

All these factors hardly affect the cleavage of soluble synthetic substrates in a comparable way in vitro, such as the cleavage of fluorescence generating 4-methylumbellyferyl containing soluble molecules, often used in the medical literature to assay the overall hydrolase activity in biological samples.

There is ample evidence that a monogenic defect can trigger a cascade of metabolic errors in lysosomal storage diseases. Besides drugs like CADs [52], the main posttranslational regulators in the endosomal and lysosomal compartment can modify the lipid cleaving activities of lysosomal hydrolases effectively [53]. The lipid composition and lipid functions of cellular membranes are cell-type and organelle specific. For example, cholesterol, SM, and gangliosides are essential stabilizers of many neuronal plasma membrane functions. In the lysosomal compartment, however, SM and cholesterol are effective inhibitors of several SAPs and of GSL catabolic pathways, including those of ganglioside GM2 and glucosylceramide [49].

**Figure 2:**
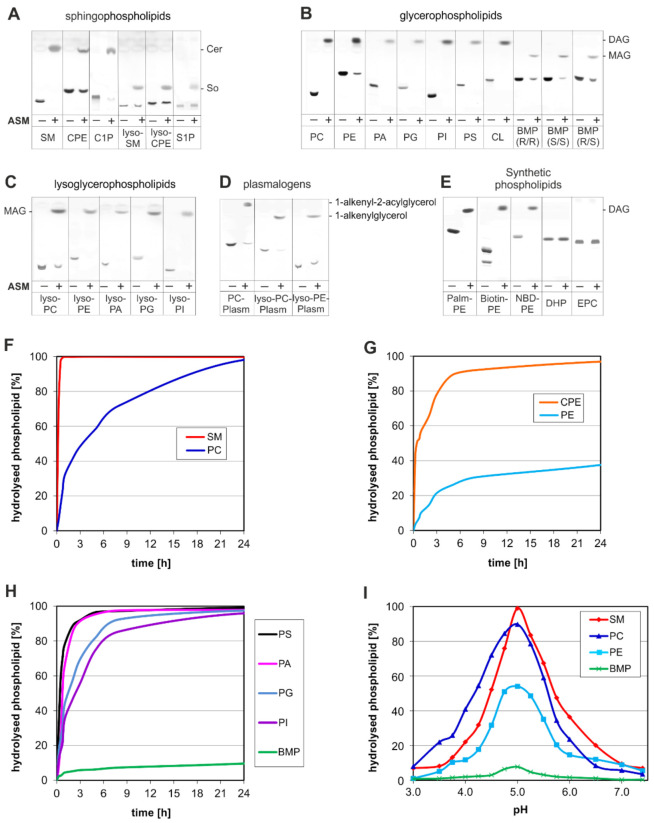
Degradation of phospholipids by recombinant ASM. The ability of ASM to hydrolyze (**A**) sphingophospholipids, (**B**) glycerophospholipids, (**C**) lysoglycerophopholipids, (**D**) plasmalogens (Plasm) and in (**E**) synthetic phospholipids (*N*-palmitoyl-dipalmitoyl phosphatidylethanolamine (Palm-PE), *N*-biotin-phosphatidylethanolamine, *N*-NBD-phosphatidylethanolamine and dicetylphosphate (DHP)) was tested in a detergent-based assay. 15 µg lipid were incubated in 20 mM sodium citrate buffer containing 0.05% Triton X 100 (pH 5) in a final volume of 100 µL with 10 µg recombinant ASM [62] for 24 h at 37 °C. The reaction was stopped by the addition of 350 µL chloroform/methanol 1:2 (*v/v*), to obtain a homogeneous phase. For lipid separation by thin-layer chromatography, the chromatograms were developed with chloroform/methanol/water (60:25:4, *v/v/v*) up to 7.5 cm and twice with n-hexane/diethylether/glacial acetic acid (70:30:1, *v/v/v*) up to the end (10 cm). To identify the hydrolysis products, standard lipids (ceramide, diacylglycerol, monoacylglycerol, palmitic acid, and sphingosine) were applied. The developed thin-layer chromatography plates were sprayed with an aqueous solution, containing 8% (*w/v*) H_3_PO_4_ (85%) and 10% (*w/v*) CuSO_4_, and charred at 180 °C for 10 min. In (**F**–**H**) time dependent and (**I**) pH dependent studies, the lipids were incubated under the same conditions as in (**A**–**E**), but by varying the time (0–24 h) or the pH values of the assay (3.5–7.4) as indicated. For quantitative analytical thin-layer chromatography determination, increasing amounts of standard lipids were applied and the plates were quantified by scanning densitometry at 595 nm. Biotin-PE: *N*-[6-(biotinoyl)amino]hexanoyl-1,2-dihexadecanoyl-*sn*-glycerol-3-phosphoethanolamine, BMP: bis(monoacylglycero)phosphate, C1P: ceramide-1-phosphate, Cer: ceramide, CL: cardiolipin, CPE: ceramide phosphoethanolamine, DAG: diacylglycerol, DHP: dihexadecyl phosphate also called dicetylphosphate, EPC: 1,2-dioleoyl-*sn*-glycero-3-ethylphosphocholine, lyso-CPE: sphingosyl phosphoethanolamine, lyso-PC-Plasm: 1-*O*-1′-(*Z*)-octadecenyl-2-hydroxy-*sn*-glycero-3-phosphocholine, lyso-PE-Plasm: 1-*O*-1′-(*Z*)-octadecenyl-2-hydroxy-*sn*-glycero-3-phosphoethanolamine, MAG: monoacylglycerol, NBD-PE: *N*-(7-nitrobenz-2-oxa-1,3-diazol-4-yl)-1,2-dihexadecanoyl-*sn*-glycero-3-phosphoethanolamine, PA: phosphatidic acid, Palm-PE: *N*-palmitoyl-phosphatidylethanolamine, PC: phosphatidylcholine, PC-Plasm: 1-(1*Z*-octadecenyl)-2-docosahexaenoyl-*sn*-glycero-3-phosphocholine, PE: phosphatidylethanolamine, PG: phosphatidylglycerol, PI: phosphatidylinositol, PS: phosphatidylserine, S1P: sphingosine-1-phosphate, SM: sphingomyelin, So: sphingosine.

The main PL-degrading phospholipases in the endolysosomal compartments are the phosphodiesterase ASM and the lysosomal phospholipase A2 [54]. It has been shown that both the secretory ASM purified from human urine [12] and the ASM purified from human tissue [55,56] not only hydrolyze SM but also various glycerol-PLs such as phosphatidylcholine (PC) and phosphatidylglycerol (PG). Already in 1980, PLC activity had been recognized in a soluble extract of rat liver lysosomes [57] and other tissue [58] with a pH optimum of pH 4.5 [57,59,60], but the enzyme has never been characterized. However, since this lysosomal PLC activity was also deficient in fibroblast extracts from patients with ASMD, its nature was assumed to be ASM [61]. To support that notion, we studied the hydrolytic capability of a recombinant ASM, thus being free of any contamination by other lysosomal hydrolases. Indeed, ASM has a much broader substrate specificity than expected so far. In a micellar assay system, ASM cleaves 27 sphingo- and glycerol-PLs: Sphingo-PLs (SM, ceramide phosphorylethanolamine, and ceramide-1-phosphate) were degraded to ceramide, lyso-sphingo-PLs (lyso-SM also called sphingosyl phosphorylcholine, lyso-ceramide phosphorylethanolamine also called sphingosyl phosphorylethanolamine, and sphingosine-1-phosphate) to sphingosine (Figure 2A). Sphingo-PLs (such as SM, ceramide phosphorylethanolamine) were hydrolyzed faster by ASM than their glycerol-PL analogues (PC and phosphorylethanolamine) (Figure 2F,G). Glycero-PLs (Figure 2B,E) were hydrolyzed to diacylglycerol, lyso-glycero-PLs (Figure 2C) and BMP to monoacylglycerol (Figure 2B), plasmalogen to 1-alkenyl-2-acylglycerol, lyso-plasmalogen to 1-alkenylglycerol (Figure 2D).

Only the artificial and synthetic PLs dicetylphosphate and 1,2-dioleoyl-*sn*-glycero-3-ethylphosphocholine were not cleaved by ASM (Figure 2E).

BMP is a unique anionic lyso-PL of the ILVs that effectively stimulates all steps of sphingolipid catabolism in the lysosome which have been analyzed so far and it contributes to generating a negative surface charge on intraendolysosomal vesicle surfaces [49]. In mice, BMP levels apparently adapt to the metabolic state in a cell type-specific manner: they are elevated in hepatocytes and pancreatic cells after fasting [63]. A slow degradation of BMP by an unspecified lysosomal phosphodiesterase activity [64] was observed earlier, which might have originated from ASM activity.

## 4. Emerging Functions of ASM, a Promiscuous Phospholipase C, Cleaving Membrane Lipids, Including Cer1P and BMP

Functional ASM is crucial to prevent not only fatal lysosomal SM storage but also a secondary accumulation of cholesterol, lyso-SM, and glycolipids such as glucosylceramide and gangliosides GM2 and GM3 as observed in ASMD [53,65]. An extensive report on clinical course and autopsy findings of a three year old male patient with neurovisceral pathology was published recently [66]. For the diagnosis of ASMD, the plasma level lyso-SM, a biomarker for ASMD, is used [65]. Accumulating SM and cholesterol can attenuate the activity of sphingolipid activator proteins and key steps in lysosomal digestion of macromolecules triggering a cascade of metabolic errors. Furthermore, ASM and the regulation of its SM-cleaving activity are important players to balance the ratio of membrane stabilizing SM and stress signaling ceramide levels properly [67]. Studies on heterozygous and thus partially ASM deficient mice and on wild type mice showed that ASM tempers leukocyte entry into the re-perfused ischemic brain [68]. In addition, experiments including ASM-deficient cells and mice identified ASM as a key player to mediate radiation-induced (a high dose of 15 Gy) apoptosis of endothelial cells [69,70]. ASM turned out to be a primary target for radiation-induced apoptosis and tumor shrinkage. These stressors are thought to effectively trigger ASM activity and thereby induce rapidly increasing cellular ceramide levels. Spatially confined increases of membrane ceramide may then lead to the reorganization of lipid rafts and thereby induce corresponding transmembrane downstream signaling [67].

However, the question remains why other major ASM-substrates of cellular membranes such as the PLs, PC and PG, and their metabolites [12,48] are usually not considered in the medical literature and in the molecular model mentioned above as possible players mediating pathophysiological consequences of varying ASM. One reason might be based on the outstanding SM accumulation compared to the relatively minor changes of the glycerol-PL pattern in ASMD. There is indeed one important difference between the catabolic pathways of the ASM substrates. ASM is the only lysosomal phosphodiesterase cleaving the sphingolipid SM, whereas the glycerol-PLs, PC, PG, and many other PLs, can also be degraded by other phospholipases in parallel. Though the lysosomal degradation of PC, PG, and many other PLs generates a greater variety of catabolites than the SM catabolism, ASM deficiency has a much higher impact on SM accumulation than on many other ASM substrates. ASM mediated cleavage of both, membrane-bound SM and PC, is stimulated by anionic PLs such as BMP, PG and phosphatidic acid whereas the lipid metabolites, diacylglycerol, ceramide and free fatty acids, stimulate SM hydrolysis but inhibit PC cleavage (Table 1) [48].

Recent data (Figure 2) underline and further specify the broader substrate affinity of ASM and characterizes ASM as a promiscuous PLC, cleaving besides SM more than 20 membrane lipid species, PLs and lyso-PLs. As depicted in Figure 2A ASM is also capable of cleaving Cer1P, a growth factor on tumor cell surfaces, most likely to the growth inhibitor ceramide. Cer1P is a membrane bound component of the cell surfaces that protects endothelial cells from apoptosis [71,72] and induces proliferation in fibroblasts [73]. Therefore, ASM has the potential to trigger apoptotic as well as proliferative signaling pathways.

An incomplete understanding and narrowed view on ASM as a SM-only specific hydrolase generating ceramide as the one and only important signaling molecule may possibly result in a misleading interpretation and finally wrong conclusion of research data.

Considering ASM’s many physiologically important PL-substrates and their catabolites, we should be cautious not to attribute ASM induced cellular processes solely to changes in SM and ceramide levels as prevailingly stated in the in the medical literature.

**Table 1:** Regulation of PL cleavage activity of ASM by lipids.

Regulating Lipid	Sphingomyelin	References	Phosphatidylcholine	References
BMP	+	[42,48]	+	[48]
Anionic PLs	+	[42,48,74]	+	[48]
Cationic lipids/drugs	−	[48]	−	[48]
Cholesterol	◯	[48]	+ (BMP) − (other anionic lipids)	[48]
Ceramide	(+)	[48]	−	[48]
Diacylglycerol	+	[12,48]	−	[48]
Fatty acids	+	[12,48]	−	[48]
Monoacylglycerol	+	[12,48]	−	[48]
Phosphatidylinositol-3,5-bisphosphate	−	[75]		
Phosphatidylinositol-4,5-bisphosphate	−	[12]		
Phosphatidylinositol-3,4,5-triphosphate	−	[76]		
Sphingosin-1-phosphate	− ^1^	[77]		

^1^ in vivo; +, stimulation; −, inhibition; ◯, no effect.

## 5. Topology and Regulation of PL-Cleaving Activity of ASM

Lysosomes have been discovered by de Duve [78] as stomachs of the cell, degrading macromolecules at low pH-values and releasing their components into the cytosol as building blocks for biosynthetic processes and as fuel for energy metabolism. They are now considered as signaling organelles controlling a wide range of cellular functions [79,80]. Complex lipids such as SM and other macromolecules can reach late endosomes and lysosomes by endocytosis [81,82] and also by autophagy and phagocytosis [47,83,84,85,86]. Plasma membrane-bound biotin- and radio-labeled ganglioside GM1 can reach luminal vesicles of late endosomes and ILVs of the lysosomal system for final degradation as observed by immuno-electron microscopy and metabolic studies [87]. Intraendosomal vesicles and ILVs, membrane-surrounded vesicles within the lumen of the lysosome, are generated by an inward budding of the endosomal perimeter membrane and budding off vesicles into the lumen of the lysosome, as mediated by ESCRT proteins [88]. The components of endosomal perimeter membranes sorted into intraluminal vesicles can be degraded by the digestive juice of late endosomes and lysosomes.

ASM is highly active in the lumen of late endosomes, with its activity peaking around pH 5 (Figure 2I). As a promiscuous PLC, ASM splits SM and many glycero-PLs, which can also be degraded to lyso-PLs by the lysosomal phospholipase A2. This is an important function to avoid a significant accumulation of glycerol-PLs in the absence of ASM in ASMD patients. We assume that in healthy tissue the combined action of ASM and phospholipase A2 easily coverts phospholipids of the outer membrane leaflet of ILVs into micelle-forming lyso-PLs, which then can solubilize and destroy structure and barrier function of the luminal vesicle membranes, generating micelles and other lipid aggregates from both membrane sheets and thus make them all available to the digestive lysosomal juice. Diacylglycerol generated by ASM mediated hydrolysis of glycero-PLs in late endosomes and lysosomes is most likely completely degraded within the acidic compartment of the cell and has probably no chance to escape into the cytosol. Diacylglycerol, however, reaching the cytosolic leaflets of cellular membranes could trigger the release of arachidonic acid from glycero-PLs, the precursor of eicosanoids, a group of local and lipophilic signaling molecules including prostaglandins, prostacyclins, thromboxanes, lipoxins, and leukotriens.

### 5.1. Lipid Sorting and Maturation of ILVs

ILVs can be easily attacked and digested by the lysosomal juice, whereas the lysosomal perimeter membrane is well protected by a thick and stable glycocalix covering its inner, luminal surface. It prevents hydrolases and lipid binding proteins, the sphingolipid activator proteins, to reach and digest the perimeter membrane. The coat is generated by heavily glycosylated integral membrane proteins with almost digestion resistant polylactosamine units [89].

On the other hand, digestion of ILVs by the lysosomal juice is facilitated considerably by a lipid sorting process leading to a maturation of ILVs mainly at the level of the late endosomes [81,90]. The removal of the stabilizing lipids SM and cholesterol originating from the plasma membrane seems to be essential to reach a physiological turnover rate for many complex glycosphingolipids at the ILVs [53]. For instance, lysosomal accumulation of SM, e.g., in ASMD, triggers a cascade of metabolic errors, first a considerable secondary accumulation of cholesterol by inhibiting its secretion as mediated by the Niemann-Pick protein type C (NPC2) [48]. Both accumulating lipids, SM and cholesterol, are effective inhibitors of several lysosomal lipid binding proteins [46,50,91,92] and key steps in lysosomal sphingolipid catabolism [46,48,93] (Table 2).

Increased SM and cholesterol levels of the intraendolysosomal vesicles (e.g., in ASMD and Niemann-Pick type C disease) inhibit the function of several sphingolipid activator proteins such as GM2AP, Sap A and B [46,50,91,92] and the catabolism of glycosphingolipids, such as ganglioside GM2 and glucosylceramide [43,46,50], which are therefore accumulating secondarily (Table 2). They probably also attenuate the function of further lipid binding proteins (besides NPC2, also Sap C and D) and catabolic steps of complex lipids accumulating in ASMD such gangliosides GM3 [94] and GM2 [95].

**Table 2:** Storage lipids (SM, cholesterol) in ASM deficiency and their hydrolysis products (ceramide, diacylglycerol) regulate cellular processes.

	Sphingomyelin ^1^	Cholesterol ^2^	Ceramide ^3^	Diacylglycerol ^4^	References
**Lipid transfer**					
Cholesterol transfer by NPC2	−		+		[48,96]
GM1 transfer by GM2AP ^5^	−	−	−		[46,50]
**Membrane solubilization by**					
Sap A		−			[92]
Sap B		−			[91]
GM2AP ^5^	−	−	+	+	[46,50]
**Lysosomal lipid degradation**					
GM2 by Hex A/GM2AP ^5^	−	−	+	+	[46,50]
GlcCer by GBA1 ^6^	−	+	+	+	[43]
SM by ASM		◯	+	+	[48]
**Further cellular processes**					
Membrane fusion	−		+	+	[97]

^1^ primary storage lipid by ASMD, ^2^ secondary storage lipid by ASMD, ^3^ hydrolysis product of SM, ^4^ hydrolysis product of glycerol-PLs, ^5^ GM2 activator protein, ^6^ β-glucocerebrosidase; +, stimulation; −, inhibition; ◯, no effect.

### 5.2. Activation and Inhibition of ASM

**Figure 3:**
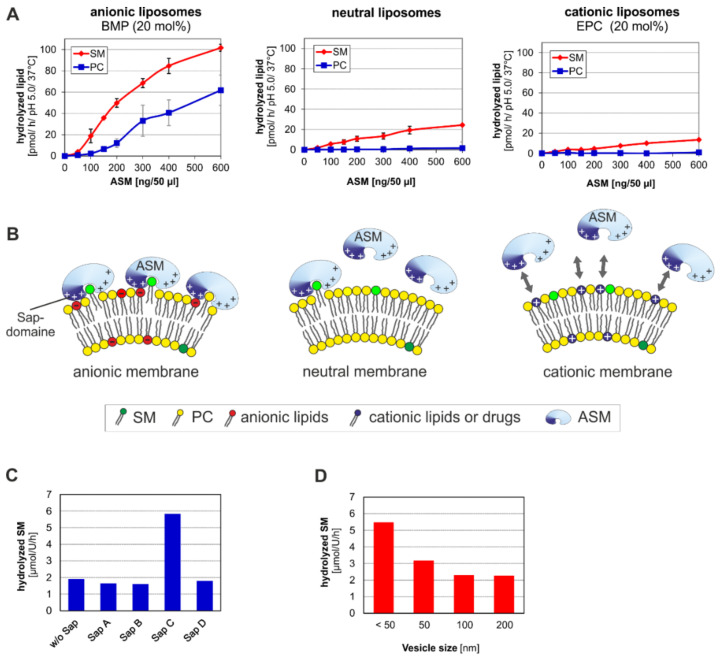
(**A**) ASM activity on membrane embedded sphingomyelin and phospholipids (e.g., phosphatidylcholine) is stimulated by anionic lipids (e.g., BMP) and inhibited by cationic substances (e.g., 1,2-dioleoyl-*sn*-glycero-3-ethylphosphocholine (EPC)) [48]. (**B**, **left**) The cartoon shows a strong electrostatic binding of cationic ASM to a negatively charged (anionic BMP containing) membrane surface, supporting an ASM mediated hydrolysis of membrane embedded sphingomyelin and other ASM-substrates. (**B**, **middle**) shows a decreasing electrostatic attraction, a reduced binding or loose attachment of cationic ASM to a neutral membrane surface reducing the catabolism of sphingomyelin and other phospholipids. (**B**, **right**) visualizes a strong electrostatic repulsion between cationic ASM and a positively charged membrane surface containing cationic lipids (EPC), effectively reducing the hydrolysis of membrane embedded SM and other phospholipids by a released ASM, which furthermore may become an easy prey of proteolytic digestion in a lysosomal environment [28]. (**B**) was modified after [48]. (**C**) External Sap C treatment and (**D**) a small vesicle size of negatively charged liposomes stimulates sphingomyelin degradation best. Data are taken from the following publications: (**A**) [48], (**C**,**D**) [42]. ASM: acid sphingomyelinase, BMP: bis(monoacylglycero)phosphate, EPC: 1,2-dioleoyl-*sn*-glycero-3-ethylphosphocholine, PC: phosphatidylcholine, SM: sphingomyelin.

The regulation of the SM-cleaving activity of ASM has been poorly analyzed so far. Due to its Sap-domain, ASM has a basic SM-cleaving activity even in the absence of lipid binding proteins. However, both Sap C [42] (Figure 3C) and Sap D [98], stimulate SM-cleavage substantially in vitro. This effect is probably caused by the fusion triggering and membrane destroying properties of Sap C and Sap D [99,100,101,102,103,104]. Mutations in the Sap-domain of ASM abrogating disulfide bridges or altering the charge are enzymatically inactive in a micellar as well as in a liposomal assay system, even in presence of external Sap C [105]. In vitro studies revealed that anionic lipids in the SM carrying membranes of liposomes boost SM and PC cleavage by ASM effectively, whereas cholesterol strongly inhibits PC but not SM-degradation [48] (Table 1). Catabolism is also directly dependent on the curvature of the SM-carrying membrane. As expected, degradation rates of SM were higher in small SM-containing vesicles (SUVs) with a high curvature than in large unilamellar vesicles (LUVs) with a low curvature [42] (Figure 3D).

Several organelle-specific phosphatidylinositolphosphates can affect ASM activity (Table 1). Its SM cleavage is inhibited by PI(3,4,5)P_3_ and by PI(4,5)P_2_, both located at the plasma membrane, or by PI(3,5)P_2_, localized at the perimeter membrane of late endosomes and lysosomes [12,75,76], whereas lipids enriched in intraendolysosomal vesicles [106,107] like phosphatidylinositol, BMP and other anionic lipids, enhance ASM activity [42,48].

Based on in vitro studies, BMP and other anionic membrane lipids generate a negative surface potential on liposomal vesicles even at low pH values, electrostatically attracting protonated lysosomal ASM and other positively charged lysosomal hydrolases to bind to the surface of ILVs in order to effectively facilitate catabolism of their vesicle bound lipid substrates (Figure 3A,B) [48,108]. This electrostatic attraction can be compensated by the incorporation of cationic lipids or CADs into the ILV membranes [48], releasing protonated hydrolases from the substrate carrying ILV membranes (Figure 3B). Endogenous cationic sphingoid bases such as sphingosine and sphinganine inhibit the catabolism of ganglioside GM2 [46], of glucosylceramide [43] at a posttranslational level and presumably also that of SM in ASMD. Many antidepressant drugs such as desipramine and other CADs or synthetic cationic lipids can act as so called “functional inhibitors of ASM” (FIASMAs) [109] (Figure 3A) which is little misleading because it incorrectly implies an ASM specific effect. Due to their physicochemical properties, CADs can easily reach and enter the acidic compartments and accumulate there up to rather high concentrations, thereby significantly increasing the luminal pH value. This in turn abrogates the electrostatic attraction of various positively charged lysosomal hydrolases to negatively charged ILV-membranes carrying the lipid-substrates to be digested (Figure 3B) [48]. Disruption of the enzyme-substrate interaction by CADs does not only reduce the catabolic rate of PL-cleavage but also leads to premature proteolysis of the released enzymes such as ASM [28,30] (Figure 3B), but also of other hydrolases such as acid ceramidase [110] and due to preliminary evidence for β-galactosidase and hexosaminidases as well.

Desipramine reduces lysosomal ASM activity in fibroblast culture almost completely, down to few percent within minutes whereas this effect can be halted completely by the preincubation of cells with a protease inhibitor like leupeptin. The more prone an ILV-associated glycoprotein is to proteolytic digestion, the faster its CAD induced depletion. ASM appears to be one of the most proteolysis sensitive proteins resulting in a very fast degradation once detached from ILVs.

Many clinically approved cationic amphiphilic drugs can accumulate in lysosomes of cancer cells. They destabilize their membranes and interfere with their cancer promoting functions opening new possibilities for cancer therapy [111].

## 6. ASM Functions within Endosomes and Lysosomes

As the only SM degrading lipase, functional ASM is essential for the avoidance of a progressive and fatal accumulation of SM and its lyso-derivative, sphingosyl phosphocholine—a mitogen [112]—as seen in ASMD. A deficiency of promiscuous ASM in ASMD type A also leads to a percentage increase of BMP in liver similar to that of SM [113], suggesting that ASM is the main enzyme catabolizing lysosomal BMP (Figure 2B,H,I). Furthermore, ASM plays a major role for the maturation of ILVs and the physiological catabolism of many glycosphingolipids.

A main function of ASM is to reduce inhibitory SM levels in intraluminal vesicles in the late endosomes and ILVs in order to establish a physiological rate of cholesterol secretion from the late endosomal compartment and to allow a normal lipid turnover at ILV membranes in the lysosomes [48,107].

Besides a secondary accumulation of cholesterol, the lysosomal SM storage effectively triggers a ganglioside GM2 and glucosylceramide accumulation in ASMD and impedes many catabolic pathways of glycosphingolipids [48,50]. Together, cholesterol and SM trigger a cascade of errors in lysosomal lipid metabolism, also impeding all lipid binding proteins tested so far, GM2AP, Sap A and B, and probably also some more. Their inhibition may well cause secondary increases of further glycosphingolipids such as ganglioside GM3 and lactosylceramide [114]. SM also inhibits vesicle fusion, which is facilitated by anionic BMP and ceramide at low pH values together with lipid binding proteins, NPC2 [48], Sap A, C. Supporting this, D. Ruiz-Argüello et al. demonstrated that the in situ generation of ceramide and diacylglycerol leads to vesicle fusion and membrane leakage [97] (Table 2).

Ceramide, generated by an ASM mediated SM breakdown, is a stimulator of many endolysosomal processes e.g., cholesterol transfer by NPC2 [96], activity of glucocerebrosidase [43] and GM2 hydrolysis by β-hexosaminidase A/GM2AP [46] (Table 2). Ceramide can eventually be converted to Cer1P by ceramide kinase (CERK) for the production of chemokines and cytokines [115]. Cer1P is a signaling lipid, which impacts in the regulation of cell growth and survival, inflammation and tumor dissemination [116]. Ceramides became known as tumor suppressor lipids that trigger apoptotic cell death by acting directly on a voltage dependent anion channel VDAC2 of mitochondria [117].

## 7. Can Secreted ASM Act at Cellular Membrane Surfaces Directly?

Cultured human fibroblasts secrete substantial amounts of an active 75 kDa ASM glycoprotein with a molecular mass of 64 kDa for the peptide chain (Figure 1C). The secretion of ASM is obviously heavily increased in patients stressed by a cancer breakthrough or heavy traumatic organ damage. Their urine served as a source for the first successful purification of ASM to homogeneity [12]. Endothelial cells of mice have been identified as the main source of ASM and ceramide secretion induced by a high, single (15 Gy) radiation dose [118], which triggers apoptosis of endothelial cells [119]. Increased ASM activity and generation of ceramide is a critical response to cell stress. For example, ASM generated ceramide is considered to be a crucial mediator of alveolar destruction in emphysema [120]. A high radiation may raise the instability of endothelial cells and loosen the structure of their membranes [121]. An in vivo assay with fluorescently labeled SM-containing liposomes can be used as a controlled release system of labelled SM in tumor diagnosis after radiation-induced cell stress [122]. The crucial role of increasing ceramide levels generated by activation of ASM during the radiation induced apoptosis of endothelial cells in the central nervous system has been described before [123,124]. However, it is unclear if ceramide is the only and sufficient player to trigger wide-spread apoptosis of endothelial cells. Considering the broad specificity of the promiscuous ASM, cleaving more than 20 PLs besides SM, it is quite likely that the digestion of several constitutional membrane components, Cer1P and the PLs, will substantially contribute to the induction of transbilayer lipid movements [125] and will affect the integrity of some cellular membranes [125]. The secretion of ASM to reach the cellular surface and the extracellular space under normal and stress conditions has been observed already in early studies [12,21,22,126,127].

Therefore, we hypothesize that ASM mediated cleavage of the growth factor Cer1P together with the ASM-triggered extensive breakdown of cellular membrane-lipids could be a plausible cause leading to apoptosis of endothelial cells as observed in tumors of the central nervous system treated with a high single radioactive dose [124].

Intriguingly, the *SMPD1* gene coding for ASM of endothelial cells is regulated at the genetic level by the microRNA, miR-15a [121]. A low dose of radiation (2 Gy) upregulates miR-15a and lowers *SMPD1* levels, whereas a high dose of radiation (more than 10 Gy) lowers miR-15a and activates the *SMPD1* gene, increasing ASM biosynthesis. Antidepressant treatment also decreases expression of *SMPD1*-mRNA [128]. In a similar manner, the NO-donor diethylenetriamine/NO also decreased mRNA and protein expression of ASM in HepG2 cells, triggering apoptosis and cell death [129].

While we know some features of the topology and regulation of ASM activity in late endosomes and lysosomes, little is known about the mechanism and function of secreted ASM acting on PLs of the plasma membrane. So far, no significant levels of ASM stimulating factors have been reported to be present at the cell surface, such as low pH values, presence of Sap C or anionic PLs like BMP. Molecular dynamics simulations and molecular docking studies suggest that at neutral pH and also at low pH below 3.0 ASM adopts a conformational fold making it inaccessible for the binding of SM at the catalytic site, in contrast to the open and accessible ASM structure identified at pH 5 [130]. Of course, local and transient molecular and biophysical changes at the plasma membrane may well overcome these activity barriers and stimulate SM and Cer1P cleavage at the cellular surface even by the secreted and soluble ASM. However, no clear evidence is available for SM and Cer1P hydrolysis being directly mediated by secreted ASM at the cellular surface, i.e., on the outer leaflet of intact plasma membranes. Alternatively, lipids of the plasma membrane may cycle back and forth through early endosomes [131], where they could be attacked by ASM at slightly lower pH-values. Secreted ASM seems to be able, at least by an indirect way, to hydrolyze sphingomyelin present at the outer leaflet of cellular membranes to ceramide [132].

On the other hand, atherogenic lipoproteins including SM have been described to be excellent substrates for secreted ASM, even at neutral pH-values. Though secreted ASM can hydrolyze native plasma LDL at pH 5.5, but not at pH 7.4, LDL modified by oxidation and phospholipase A2 treatment has been reported to be readily hydrolyzed by secreted ASM even at pH 7.4, as reported for atherosclerotic lesions [133].

## 8. Medical Importance of Cellular ASM Activity

ASM is an essential player in the cellular PL metabolism and key for the maturation of intraendolysosomal luminal vesicles. It is a promiscuous lysosomal and secretory PLC, the PL-cleaving activity of which is subject to complex regulation at the genetic and posttranslational level. Changes in ASM activity affect a wide field of the cellular lipid metabolism and may control the fate of the cell, especially the levels of SM and ceramides and in part also the turnover of many other PLs and lyso-PLs. Inherited ASMD, but also variations of ASM activity as controlled by posttranslational regulation, can contribute to neurodegenerative and systemic disorders. They may change the cellular and organellar balance between SM and ceramide levels and modify the PL and lipid pattern of many membranes in a cell-specific manner. The underlying regulatory mechanisms are only poorly understood. Since lysosomal sphingolipid catabolism follows a strictly sequential and stepwise pathway and ASM is the only lysosomal phospholipase degrading SM (and possibly also the sphingolipids ceramide phosphoethanolamine, lyso-SM, and lyso-ceramide phosphoethanolamine), its activity has a direct impact on the molecular ratio between SM and ceramide. This is obviously different for many glycerol-PLs, lyso-glycero-PLs and plasmalogens degraded by ASM as given Figure 2A–E. The catabolism of glycerol-PLs usually does not follow a single, exclusive pathway. They can be degraded by different phospholipases, ergo by multiple pathways simultaneously. In other words, if one pathway is deficient, a bypass can compensate at least partially. Nevertheless, it is expected that a downregulation of ASM activity will not only affect cellular SM and ceramide levels, but to some extent also glycerol-PLs and their pattern in cellular membranes.

ASM activity is also important for several intracellular signaling pathways, for example the chemotherapy induced generation of ROS injury [134], the promotion of ferroptosis [135] and the vascular calcification [136].

The complex regulation of PL-cleaving ASM activity can hardly be mimicked by in vitro assays [42]. In vitro assays do not reflect the in vivo lipid cleaving activity of a hydrolase, especially if a soluble synthetic substrate is used. For example, the hydrolysis of soluble 4-methylumbelliferyl-6-sulfo-2-acetamido-2-deoxy-β-d-glycopyranoside (MUGS) by β-hexosaminidase A is not affected by effective stimulators and inhibitors of its ganglioside GM2 cleaving activity [46]. Furthermore, CADs and storage compounds accumulating in lysosomal diseases can inhibit GM2 breakdown effectively, but hardly the hydrolysis of soluble substrates like MUGS in vitro [52,53,93].

## 9. Assays for Studies of ASM Inhibitors

In vitro ASM activity assays can demonstrate the presence of ASM in biological samples quantitatively, but they cannot determine its lipid substrate degrading potential in vivo (SM, PC, Cer1P, phosphatidylethanolamine and many others). However, the use of ASM specific inhibitors, e.g., in cultured cells followed by a lipid analysis mass spectrometry can at least identify and quantify lipid-levels affected by reduced ASM activity.

In cell culture, CADs such as desipramine, chloroquine, propranolol and many others, reduce the lipid cleaving activity of ASM effectively, and that of other lysosomal hydrolases [28,52]. This decline in lipid cleaving activity, however, is neither caused by direct inhibition of the ASM enzyme nor is it specific for ASM. As described in Section 5.2, CADs reduce the electrostatic attraction between protonated and positively charged lysosomal hydrolases and their sphingolipid-substrate carrying ILV-membranes. Based on their physicochemical properties, CADs concentrate in ILV-membranes, decreasing or even abolishing their negative surface potential, needed to attract the catabolic enzymes and protein cofactors.

On the other hand, the development of specific ASM small molecule inhibitors has made great progress recently. Together with a genetic silencing of the *SMPD1* gene coding for ASM, they may help to develop tools for analyzing changes of cellular lipid and PL pattern caused by a reduction of ASM activity in more detail.

Unfortunately, SM has been considered in the literature to be the only PL substrate of ASM and together with its product ceramide to be the only membrane lipid affected directly by changes of ASM activity, despite the broad substrate spectrum observed in vitro, identifying ASM as a promiscuous PLC (Figure 2). An ASM mediated breakdown of major glycerol-PLs like PC, phosphatidylethanolamine, phosphatidylserine, phosphatidylinositol, PG, phosphatidic acid, BMP and many more as observed in vitro (Figure 2) should labilize the bilayer structure of intraendolysosomal luminal membranes and allow the digestion of lipid components of the inner leaflet of intraendolysosomal vesicles within the lysosomal compartment. The broad spectrum of ASM substrates as defined in vitro (Figure 2) strongly suggests the digestion of the signaling PL Cer1P and others such as ceramide phosphoethanolamine, lyso-SM, lyso-ceramide phosphoethanolamine, and of many glycerol-PLs in vivo by ASM (Figure 2).

In order to study the role of ASM in diseases such as sepsis, acute lung injury or metastasis of melanoma cells, a promising and specific FRET (Förster resonance energy transfer) based assay has been developed to monitor ASM activity in living cells [137,138,139]. Recently, the same group also developed a photocaged inhibitor of ASM with *O*-nitrophenyl photocages and butyryl esters to mask hydroxyl groups transiently. The potent light-inducible inhibitor allows a time-resolved inhibition of ASM activity in intact living cells [140].

An interesting two-component liposome-based colorimetric assay has been described for a neutral bacterial sphingomyelinase. It is rather fast, specific, and more sensitive than currently available commercial tests [141]. It is therefore suitable for a rapid screening of sphingomyelinase inhibitors and can probably easily be adjusted to also screen inhibitors of human ASM at low pH-values.

Small functional inhibitors of ASM have been described [142,143,144]. A novel and selective small inhibitor for ASM, a *N*-hydroxy-3-alkoxybenzamide, showed anti-apoptotic and anti-inflammatory activity. It presumably binds key residues and the Zn^2+^ cofactor of the ASM protein [145]. Another direct inhibitor of ASM (compound **21b**) was developed to improve depression-like behaviors of rats [146]. Apparently Avicin G is a potent inhibitor of both, the neutral sphingomyelinase of the plasma membrane and the lysosomal ASM. It depletes both, phosphatidylserine and cholesterol, from the inner leaflet the plasma membrane and mis-localizes K-Ras [147]. An interesting, apparently active site directed small molecule is ARC39 (1-aminodecylidene-bisphosphonic acid), which inhibits lysosomal ASM in intact living cells [148]. The transfusion of ARC39-treated or ASM-deficient aged platelets have lower ceramide levels and alleviate lung injury, compared to wild-type platelets. ASM is also associated with the pathogenesis of lung diseases such as cystic fibrosis and acute lung injury, since ASM deficient mice showed better lung mechanics and were protected from bronchial asthma TH2-regulated allergic bronchial asthma model [149].

The baseline of cellular ASM activity is important to maintain and regulate lysosomal functions properly. ASM is not only needed to allow maturation of ILVs by degrading inhibitory SM and thereby facilitate the secretion of inhibitory cholesterol from the lysosome as discussed above in Section 5, but also functions to maintain physiological mTOR signaling and to inhibit autophagy. The downregulation of ASM activity reduces and modifies signaling by the lysosomal nutrient-sensing complex (LYNUS), increases autophagy and decreases the levels of sphingosine and sphingosine-1-phosphate [150]. Antidepressant drugs and CADs such as the “functional ASM inhibitors” fluoxetine and amitriptyline, can trigger a slow accumulation of SM, cholesterol and other lipids in the lysosome, and induce autophagy (Figure 1C) for instance in hippocampal neurons [151] and furthermore in glioma cells by pimozide and loperamide. Recently, it was shown that ASM plays an important role in ferroptosis, an iron-dependent cell death which is genetically and biochemically distinct from other forms of regulated cell death [135].

ASM also plays a major role in the development of many other disorders such as cardiovascular and metabolic diseases. Its downregulation was reported to alleviate vascular endothelial insulin resistance in diabetic rats [152] and improve vascular dysfunction in diabetic mice [153]. Amitriptyline inhibits nonalcoholic steatohepatitis and atherosclerosis as induced by high-fat diet and lipopolysaccharide in mice [154]. The survival of cultured oligodendrocytes treated with glutamate was enhanced by the downregulation of ASM-expression as well as by blocking ASM activity [155]. Partial genetic inhibition of the increased ASM levels observed in cultured neurons derived from familial Alzheimer patient with lysosomal depletion restored an autophagic dysfunction by recovering lysosomal biogenesis [156]. Inhibition of upregulated ASM in primary multiple myeloma cells by the functional inhibitor amitriptyline increased the sensitivity to antimyeloma drugs [157]. Upregulation of SM turnover in isolated neuronal plasma membranes may also play a critical role in the action of general anesthetics. The treatment of isolated nerve ending membranes with halothane or xenon gas increased the hydrolysis of membrane-bound SM by membrane-bound (presumably neutral) sphingomyelinase at neutral pH-values coinciding with increasing membrane fluidity. Already, clinical concentrations of halothane increased SM hydrolysis significantly, and this effect could be enhanced further up to 10-fold by raising halothane concentrations [158]. General anesthetics such as halothane and N_2_O-gas also increase the breakdown of membrane-bound ganglioside GD1a by membrane-bound sialidase in parallel to increasing membrane fluidity [159].

## 10. Major Depression and the Emerging Role of ASM and Acid Ceramidase in Lysosomal Lipid Turnover

Major depressive disorder is a global contributor to disability and mortality. The proposed mechanisms of depression are many-fold but poorly established. Antidepressant drugs such as desipramine, fluoxetine, a serotonin reuptake inhibitor, and amitriptyline are thought to increase serotonergic and other neurotransmitter levels in the synaptic cleft of nerve endings. They and many other antidepressant drugs are amphiphilic lipid-like and positively charged CADs, that can concentrate in luminal vesicles of the endolysosome and compensate their negative surface charge. Thereby, positively charged lysosomal hydrolases like ASM, acid ceramidase, hexosaminidases and many others are released and separated from their substrate-carrying membranes and become proteolytically degraded by the lysosomal juice. The breakdown of the electrostatic attraction between positively charged ASM, acid ceramidase, hexosaminidases, and other lysosomal catabolic proteins and their lipid-substrate carrying ILVs, triggers a cascade of functional inhibition of the lysosomal catabolism of SM, ceramide and many other membrane lipids [47,52,53,93]. Aberrant levels of ceramide and other sphingolipids have been observed in few cases of depression [160]. On the other hand, antidepressant CADs can trigger a slow increase of lysosomal SM levels and of sometimes lipotoxic ceramide levels in ER-membranes inducing autophagy, especially in hippocampal neurons [151]. Fluoxetine increases hippocampal neurogenesis [161], amitriptyline, and fluoxetine support neuronal proliferation which is mainly mediated by a functional attenuation of lysosomal turnover of SM and other lipids [162,163]. A chronic administration of the serotonin reuptake inhibitor paroxetine and the noradrenaline reuptake inhibitor desipramine reduced sphingosine levels in the prefrontal cortex and paroxetine reduced sphingosine and ceramide levels in the hippocampus, but not sphingosine in the plasma samples. At the genetic level, the drug-induced decrease of small sphingolipids such as sphingosine and ceramide, coincided with a reduction of mRNA levels of the *SMPD1* gene for ASM and the *ASAH1* gene for acid ceramidase [164]. The inhibition of ASM, and therefore an expected slow increase of the SM level, seems to be also an important effect of many antidepressants, a view supported by findings obtained with a newly developed specific ASM inhibitor. Its administration improved depression-like behaviors in rats and increased hippocampal neurogenesis [146]. On the other hand, the overexpression of ASM in the forebrain of male mice caused a depressive-like phenotype, whereas in female mice, ASM overexpression triggered a social anxiogenic-like behavior [165]. Supporting this, possibly the activities of both sphingomyelinases and ceramidases were elevated in the brain regions of female rats selectively bred for high versus low anxiety-like behavior. High anxiety rats exhibited increased activity of ASM and neutral sphingomyelinase as well as of acid ceramidase and neutral ceramidase in multiple brain regions associated with anxiety and depressive-like behavior [166]. The functional ASM-ceramide pathway in the rat brain has also been implicated in the extinction of learned behavior—coincided with a reduction of ASM activity and decline of ceramides in dorsal hippocampus—and expression of re-learning-related behavior [167].

ASM levels increase with stress, age, and bacterial infections [168] and the downregulation of the ASM gene *SMPD1* is associated with resistance to chemotherapy regimens [169]. An indirect functional inhibition of ASM phospholipid-cleaving activity by the CAD amitriptyline prevented harmful behavioral and neurologic effects of repetitive mild traumatic brain injury in mice [163].

## 11. Experimental Therapeutic Approaches for Niemann-Pick Disease Types A and B

Since the pioneering work of R.O. Brady on enzyme replacement therapy (ERT) of patients with adult Gaucher disease, this and few other lysosomal storage diseases not affecting the brain can be successfully treated by this approach [170]. High doses of therapeutic enzyme activity are often applied, in order to reach and to digest storage material in most, even hardly accessible places of the body except those beyond the blood/brain barrier. An ERT with recombinant ASM, olipudase alfa, as treatment option for patients with ASMD type B or neurovisceral type A/B is under development. The most recently presented data from the ASCEND and ASCEND-peds phase II/III trials show a significant improvement of lung diffusion capacity (DL_CO_) as well as spleen volume reduction compared to the placebo [171]. However, since enzyme replacement therapy with high dose ASM in ASMD patients may cause toxic side effects by abruptly changing the cellular balance between SM, ceramide, and other PLs, a dose escalation strategy with olipudase alfa has been successfully developed [172,173]. Another interesting experimental approach to improve targeting and dosing was published recently [174]. Here, the therapeutic recombinant human ASM was loaded into liposomal formulations which reduced the accumulation of toxic lyso-SM in Niemann-Pick disease type B fibroblasts by 71%.

However, whether recombinant human ASM also changes levels and pattern of other sphingolipid and glycerol-PL substrates significantly and thereby affects other cellular processes is currently unknown. Of special interest are known ASM-substrates such as BMP, Cer1P, cardiolipin, ceramide phosphorylcholine, lyso-ceramide phosphorylcholine, phosphatidic acid, lyso-phosphatidic acid, lyso-SM, PC, lyso-PC, phosphatidylethanolamine, lyso-phosphatidylethanolamine, PG, lyso-PG, phosphatidylinositol, lyso-phosphatidylinositol, and phosphatidylserine.

## 12. The Role of ASM in Bacterial, Mycobacterial, Fungal, and Viral Infections

The generation of ceramide at the expense of SM by the sphingomyelinases, ASM, and neutral sphingomyelinase, is a key factor for the internalization, damaging, and killing of diverse pathogens, bacteria, and mycobacteria, such as *Staphylococcus-aureus*, *Chlamydia-trachomatis*, *Neisseria-meningitis*, *Listeria-monocytogenes*, and *Pseudomonas-aeruginosa*, *Neisseria-gonorrhoeae*, and has been reviewed recently [175,176]. ASM also contributes to the control of *Mycobacterium bovis* Bacillus Calmette-Guerin infection of murine macrophages [177]. On the other hand, the functional inhibition of ASM activity stops infection cycles of vacuole adapted bacteria such as *Anaplasma-phagocytophilum*, *Chlamydia trachomatis*, and *Chlamydia pneumoniae* or even kills *Coxiella burnettii* [109,178]. ASM is also an essential regulator of mucosal immunity to enteric pathogens. Mice with ASM deficiency or functional inhibition of ASM were highly susceptible to an infection by a *Citrobacter rodentium*-driven colitis [179].

Fungal infections are a global health threat with high morbidity. Recent discoveries were reviewed by Guzman et al. [180]. They shed insight on the mechanisms of fungal infections and on host lipids involved, including SM and ceramide and their role in phagocytotic uptake and clearance of fungal pathogens. The cellular mechanism of pathogen internalization, followed by inflammatory signaling and induced fibrosis are reviewed in [181].

Also, enveloped viruses often enter cells by endocytic pathways [182], such as rhinovirus, influenza A virus, Ebola virus, Lassa virus, and in part also SARS-CoV-2. The acidic pH of the endolysosomal compartment is crucial for the release of the viral RNA into the host cytosol [182]. It has been demonstrated that ASM activity as well as the SM and cholesterol homeostasis are important factors of the infection process. In the case of an influenza A virus infection, the accumulation of cholesterol in the endolysosomal compartment contributes to the interferon-induced attenuation of viral RNA release into the host cytosol [183]. An increased cholesterol level, either due to a primarily increased SM level or another reason, can also impair the infection by the Ebola virus and the novel virus SARS-CoV-2 [184,185,186]. Hence, it is not surprising that SARS-CoV-2 is inhibited by cationic amphiphilic antidepressant drugs, such as amitriptyline and fluoxetine, in vitro [187,188,189]. Both inhibit the endolysosomal cleavage of SM by ASM. This effect can also be observed for a lot of other viruses [190,191]. CADs seem to be promising tools to develop antiviral drugs. Unfortunately, such in vitro observations often cannot be confirmed by in vivo studies. However, in the case of SARS-CoV-2 infection the use of antidepressant CADs reduced the risk of intubation or death in hospitalized COVID-19 patients [192,193].

It has been furthermore suggested that the unique anionic lysosomal lipid BMP plays an important role in viral infections [194] and that the secretory ASM is required for the virus entry at the plasma membranes [190], reducing their high SM levels and leading to an internalization of the virus [195]. It has also been shown that sphingosine could be exploited as an antiviral compound. In the case of SARS-CoV-2-infection, sphingosine binds to the ACE2 receptor and prevents the interaction of the virus with the cellular receptor [196].

## 13. Conclusions and Perspectives

ASM appears to be the predominant lysosomal PLC. It cleaves SM along with many other PLs at surfaces of luminal intraendolysosomal vesicles and is key for the PL degradation in late endosomes and lysosome. The PL-cleaving activity of ASM is stimulated by Sap C [42] (Figure 3C) and by Sap D [98] and by anionic lipids of the substrate carrying luminal vesicles such as BMP (Figure 3, Table 1). In ASMD and few other LSDs such as mucopolysaccharidoses (Hurler, Hunter, Sly, and Sanfilippo syndrome) catabolic pathways can be inhibited by primary lysosomal storage compounds, which often trigger a cascade of secondarily accumulating lipids, aggravating the clinical course of the disease.

The hydrolysis of membrane-stabilizing SM is key for lysosomal lipid sorting and lipid digestion. An inherited deficiency of ASM in ASMD causes a major SM accumulation, which triggers a cascade of catabolic dysfunction. Accumulating SM in late endosomes and lysosomes blocks cholesterol secretion from late endosomes by inhibiting the cholesterol transfer protein NPC2, even in the presence of anionic lipids such as BMP [48,96], leading to a massive secondary accumulation of cholesterol in ASMD [197]. Both SM and cholesterol inhibit various lysosomal processes, whereas the degradation products of ASM action (ceramide and diacylglycerol) are stimulators for many lysosomal and cellular processes (see Table 2). The PL-cleaving activity of ASM controls the balance of the SM and ceramide ratio to a major extent in many cellular membranes. As secreted PLC, ASM is furthermore considered to turnover PLs of extracytosolic membrane-leaflets, including cellular surfaces. Therefore, ASM is an important player in cellular homeostasis. An unbalanced SM/ ceramide ratio can lead to cell stress and apoptosis.

The treatment of cultured cells and animals with CADs leads to an accumulation of undegraded lipids in ILVs. Based to their physicochemical properties, CADs reach luminal vesicles in late endosomes and lysosomes, compensate their negative surface charge, and reduce the electrostatic attraction of positively charged hydrolases to the membranes of ILVs. Hydrolases like ASM can be released from the ILV surfaces into the lumen of the lysosomes where they are easily degraded like ASM by lysosomal proteases [28,48]. The digestion of ASM should lead to even higher levels of SM, which may disturb membrane homeostasis and cause lysosome membrane permeabilization, which triggers cell-death by apoptosis and apoptosis-like pathways.

Due to the broad specificity of the promiscuous ASM towards sphingo-PLs and glycero-PLs, we propose the hypothesis that secretory ASM also plays an important role by cleaving the growth factor Cer1P. This, together with the massive release of ASM from endothelial cells, could explain the apoptotic cell death of tumors of the central nervous system after a single treatment with a high radioactive dose. ASM substrates such as sphingo-PLs and glycero-PLs including their lyso-derivatives and ASM mediated lipid products such as ceramide and diacylglycerol, play a substantial role in metabolic pathways of medical importance, especially in major depression, in bacterial and fungal infections, and in the regulation of lysosomal catabolism.

The molecular mechanisms regulating ASM activity at membranes of subcellular organelles are only poorly understood. Multiple stimuli regulating ASM activity are known, such as lipid composition of organellar membranes, direct oxidation of membrane components, irradiation, bacterial and viral infection, proteolytic digestion of proteins, etc.

We hypothesize that membrane lipids are not only simple building blocks of organellar membranes—like bricks of a wall—but that many of them have rather important regulator and signaling functions for metabolic pathways occurring at organellar membranes and within the organellar lumen as discussed above for the generation of secondary storage compounds by primarily accumulating membrane lipids (e.g., primarily accumulating SM triggers cascades of secondarily accumulating lipids, cholesterol, ganglioside GM2, glucosylceramide and many others). They are supposed to regulate and modify activities of organellar proteins, e.g., of enzymes and receptors. Pathological changes of the cellular lipid composition may well mis-regulate cellular metabolism, especially lipid and membrane metabolism in obesity, Alzheimer and Parkinson disease. Therefore, lipid pattern of organelle membranes should be analyzed in situ, with high spatial and temporal resolution, which is currently not possible. The currently available techniques for lipidomics of tissues and cultured cells are of little value due to their insufficient spatial resolution. Data obtained on cellular lipid distribution are averaged over many quite different submicroscopic organellar structures. Future developments will hopefully bring tools to determine organelle-specific lipid patterns and elucidate the specific regulatory functions of membrane composition.

## Data Availability

Not applicable.

## Abbreviations

ASM	Acid sphingomyelinase
ASMD	Acid sphingomyelinase deficiency
BMP	Bis(monoacylglycero)phosphate
CAD	Cationic amphiphilic drugs
Cer1P	Ceramide-1-phosphate
GM2AP	GM2 activator protein
ILV	Intralysosomal luminal vesicles
NPC2	Niemann-Pick disease protein C type 2
PC	Phosphatidylcholine
PG	Phosphatidylglycerol
PL	Phospholipid
PLC	Phospholipase C
Sap	Saposin
SM	Sphingomyelin
